# Case of Chronic Nasal Catarrh

**Published:** 1871-11

**Authors:** 


### CASE OF CHRONIC NASAL CATARRH.

Dr. Norton Folsom, Physician to the New York Dispensary, writes to the Medical Gazette:

"The following case is reported mainly for the purpose of calling attention to the convenience of the apparatus employed in treatment.

Mr. B——, æt. 35, a fine singer, free, so far as known, from any constitutional taint, had suffered for over a year from an offensive purulent discharge from the nose, which frequently formed crusts as large as the thumb, so hard and so closely adherent as to be disengaged with considerable difficulty. The voice was so much affected that singing had been almost entirely relinquished, and the fœtor of the discharge interfered with his social relations.

Rhinoscopic and anterior nasal examination showed the mucous membrane generally engorged, eroded in patches, covered with viscid muco-purulent secretion, and the lower and posterior part of the vomer entirely gone.

The nasal douche had previously been tried without benefit, but its use was resumed with a solution of permanganate of potassa, together with the application of spray of alum and of tanno-glyceine. After a few weeks, the only material improvement being the diminution of fœtor, the following line of treatment (mainly that lately recommended by Drs. Sass and Lincoln, at the Med. Lib. and Jour. Assoc.) was adopted. The whole nasal cavity being strongly illuminated with the concave mirror, with the use of the rhinoscopic mirror behind, and with a nasal speculum, contrived for the purpose, in front, the cavity was entirely freed from crusts and secretion by forceps and probes, and by the patient's own efforts with a basin of water. A solution of nitrate of silver (gr. 40–60 ad unc.) was then applied in the form of spray, from front and rear, to every part of the cavity, and the thoroughness of its action verified by examination. This was repeated at intervals of a few days for about ten weeks, the improvement being constant, and after an interval of a month, during which he grew worse, it was resumed for six weeks, when it was entirely abandoned, after about twenty applications in all, there being no offensive discharge, no formation of crusts, and the mucous membrane presenting a healthy pink appearance throughout. The voice was entirely restored. There has been no relapse during the year which has ensued.

The spray was applied with the ordinary hand-ball apparatus, the fluid being contained in a test-tube held in the hand, and the issue of the spray being instantly and completely controlled by the thumb compressing the rubber tube where it joins the atomizer. For the posterior nares the upward jet atomizer was used, a small piece of hard rubber being fitted to the tube just in front of the orifice, projecting upward about ⅜ inch, forming a palate-hook.

The addition of a few drops of *eau de Cologne* to the spray solution rendered it less disagreeable, and the after-taste was sensibly diminished by gargling with salt and water. The nostrils and upper lip were protected with an unguent.

The nasal speculum contrived for the exigencies of this case is made by coiling a piece of German silver wire at its middle, (as in an ordinary eyelid retractor), so that the ends tend to spring apart; the extremities being then bent nearly at a right angle, are curled up into blades about 1¼ inches long and ⅜ inch wide, which *flare apart* a little at the tips, which are to be introduced into the nostril. The degree of expansion is limited by a screw. The whole instrument is gilded. It is made by Messrs. Tiemann & Co. A useful addition is a piece of flexible wire attached to the ring of the instrument, which can be made to rest on the lip or cheek of the patient, and tilt or prop the nostril up horizontally. This leaves both hands free for manipulation, while the light is thrown in from the mirror on the forehead. In this way nearly every part of the naso-pharyngeal cavity can be reached, and accumulations, even upon the posterior wall of the pharynx be detached through the anterior nares.—*Med. and Sur. Reporter.*

A Remedy for Hæmoptysis.—Dr. Holden, of Newark, N. J., thus describes a method of treating Hæmoptysis, which has been successful in has practice:

"I would like to call the attention of the profession to a method of treatment of hæmoptysis, which, while most simple and efficacious, I have not seen described by any, viz: the throwing of the atomized vapor of a saturated solution of gallic acid directly into the mouth and throat. I have repeatedly found the most gratifying success follow at once, even in cases of profuse hæmorrhage. Unlike other styptics thus administered, it quiets the spasmodic cough, which seems the direct result of the presence of the blood, requires but a moment to prepare, and, aside from its efficacy, it inspires immediately the confidence of the patient. For about two years, I have adopted this method, and have been surprised that no similar experience has found its way into the medical journals. My habit has been to have an atomizer and bottle of gallic acid always at hand, and when summoned hastily, to mix the acid in a tumbler of cold water, and use even without waiting for the excess of acid to subside. It has proved successful in several cases where the blood was streaming from the mouth with every expiration."—*New York Medical Record.*

